# The Book World of Medicine and Science

**Published:** 1901-05-04

**Authors:** 


### The Book World of Medicine and Science.

The Syphilis of Children in Every-day Practice (Medical Monograph Series, No. 4). By George Carpenter, M.D.Lond. (London: Baillière, Tindal, and Cox. 1901. Price 3s. 6d. net.)

This does not strike one as being a particularly well-balanced work. The object of the series of which it forms one number is to sketch in brief compass the chief features of given subjects of every-day interest to students and practitioners, an object which hardly admits of the introduction of many clinical details. Dr. Carpenter, however, has written a work on the syphilis of children, which he has largely illustrated with examples drawn from his own work, and thus, good as it is an essay, it is not perhaps equally good as a general review of present knowledge. After a short introduction a detailed account is given of the manifestations of syphilis as this disease shows itself in the various organs and tissues of the body. A good account is given of Parrot's Nodes and Craniotabes, and of the question how these conditions are to be referred to syphilis or to rickets is discussed. In regard to cranial bosses the writer is inclined to the view that the affection is a syphilitic manifestation often occurring in a rickety subject, and that its advent is favoured by the coexistence of rickets even though it may not be very marked in degree. The supposition that the manifestation is purely rickety is not tenable. Craniotabes, he thinks, may be an evidence of rickets apart from syphilis, but he has collected statistics which show in how large a proportion of cases the disease was connected with syphilis; and he considers that the presence of craniotabes in a child should excite a strong suspicion of syphilis. When we come to the subject of treatment we are rather surprised to find Dr. Carpenter giving the first place to internal treatment with mercury and chalk, even with its acknowledged tendency to produce diarrhœa, and recommending inunction only when treatment is by the mouth is impracticable from this cause. Many useful directions are given in regard to the treatment of the various local manifestations of syphilis. We confess to a certain degree of surprise at the dose of perchloride of mercury which he says may be safely given to a child of two years old, namely one drachm of the liquor hydrarg. perchlor. with five grains of iodide of potassium three times a day. Perhaps, however, the printer may be responsible for this, as the amount of water in which it is to be taken is given as "ʒp."—a symbol which, to our minds, is not enlightening. This book contains many illustrations.

On the Use of Massage and Early Passive Movements in Recent Fractures and other Common Surgical Injuries, and the Treatment of Internal Derangements of the Knee-joint. By W. H. Bennett, F.R.C.S., Senior Surgeon, St. George's Hospital. Pp. 97, with 12 illustrations. (London Longmans, Green, and Co. 1900.)

The arguments used in this little volume are logical and convincing. The illustrations are striking and of great interest. The facts recorded are those which indicate extensive clinical experience and a close study of the subjects dealt with. Perhaps the most valuable part of the book is that dealing with internal derangements of the knee-joint. After having treated no fewer than 253 cases placed in this category, the author has a right to speak authoritatively on the matter. He states that he believes the train of symptoms so generally met with in such instances is dependent upon one of four conditions: (1) The displacement of a semilunar cartilage; (2) the nipping of folds or shreds of synovial membrane; (3) loose or pedunculated bodies in the joint; and (4) bruising of the peripheral edge of a semilunar cartilage and its attachments without actual loosening, followed by a local effusion of blood and inflammatory exudation, these insinuating themselves in between the articular surfaces. He avers that he thinks this pathological condition has not previously been described. Treatment he reviews under three headings: by rest, massage, and exercise, the use of apparatus, and operative measures. The first line of treatment is very carefully detailed and well repays perusal. In operating the author thinks that anything less than removal of the offending structure is useless. Mr. Bennett is fairly sanguine as to the success following upon operations for internal derangement, though he believes that a large share of the satisfactory terminations is due to the proper management before and after operative procedures. He believes that to operate in the presence of fluid within the joint is reckless. Possibly not all surgeons will agree with the rather limited application of exploration of the joint that he lays down, but they cannot but be convinced as to the soundness of the reasoning for his own line of practice. The author's clear and thoughtful exposition of the subjects that he treats in this little volume should be widely read and pondered over.

The Tea We Drink. By E. H. Skrine and George Browden, F.C.S. (London: Simpkin, Marshall, Hamilton, Kent & Co. 1901. Price 1s.)

About the difficulty of getting good tea we are all agreed, and about the badness of much that is on the market there can be no question. The authors of this little pamphlet show how this happens. Partly from lack of care and a desire to economise labour in the gathering, partly from difficulties in the drying of large quantities of leaves at the proper speed, and partly from the inefficiency of the machinery by which the rolling of the leaf is performed, a not inconsiderable proportion of the tea undergoes partial decomposition before the process of manufacture is completed, and this admixture of rotten leaf taints and dulls the flavour of the whole and causes various evil consequences to those who drink the product. The object of the pamphlet is to urge that as Government derives a large revenue from tea they should submit it to examination, and should give some sort of guarantee that the stuff sold to the public is at least sound. Of taste and flavour the public must judge for itself; but it is urged that rotten tea can be at once detected by an expert, and that all such should be excluded from the market. This would seem a reasonable request, especially if it is true, as is stated, that there need only be about threepence a pound difference between the good and the bad.

How to Avoid Tubercle. By Tucker Wise, M.D. (London: Baillière, Tindal and Cox. Illustrated. Pp. 24. Price, 1s. cloth; 6d. paper.)

From Dr. Tucker Wise's little tract the man in the street may well learn "to avoid tubercle." But if he follows Dr. Wise's very explicit and admirable suggestions, he will "avoid" many bacilli besides the B. tuberculosis. For after all, "the way to avoid tubercle" is to live a healthy life, following the gospel of fresh air, light, cleanliness, and sobriety. Dr. Wise's little book—for which we have nothing but praise—might well therefore have been called "How to avoid disease." It is brightly and clearly written, and well printed, the chief points being emphasised by the use of bold and heavy type. The price places it within reach of all who may care to profit by its teachings.

